# Exploring the Formulation and Approaches of Injectable Hydrogels Utilizing Hyaluronic Acid in Biomedical Uses

**DOI:** 10.1155/2024/3869387

**Published:** 2024-05-27

**Authors:** Hadeia Mashaqbeh, Batool Al-Ghzawi, Fatima BaniAmer

**Affiliations:** ^1^Department of Pharmaceutics and Pharmaceutical Technology, Faculty of Pharmacy, Yarmouk University, Irbid, Jordan; ^2^Department of Pharmaceutical Technology, Faculty of Pharmacy, Jordan University of Science and Technology, Irbid, Jordan

## Abstract

The characteristics of injectable hydrogels make them a prime contender for various biomedical applications. Hyaluronic acid is an essential component of the matrix surrounding the cells; moreover, hyaluronic acid's structural and biochemical characteristics entice researchers to develop injectable hydrogels for various applications. However, due to its poor mechanical properties, several strategies are used to produce injectable hyaluronic acid hydrogel. This review summarizes published studies on the production of injectable hydrogels based on hyaluronic acid polysaccharide polymers and the biomedical field's applications for these hydrogel systems. Hyaluronic acid-based hydrogels are divided into two categories based on their injectability mechanisms: in situ-forming injectable hydrogels and shear-thinning injectable hydrogels. Many crosslinking methods are used to create injectable hydrogels; chemical crosslinking techniques are the most frequently investigated technique. Hybrid injectable hydrogel systems are widely investigated by blending hyaluronic acid with other polymers or nanoparticulate systems. Injectable hyaluronic acid hydrogels were thoroughly investigated and proven to demonstrate potential in various medical fields, including delivering drugs and cells, tissue repair, and wound dressings.

## 1. Introduction

Hyaluronic acid is found naturally and comprises repeated negatively charged acetyl glucosamine and glucuronic units linked by 1,4 and 1,3-glycosidic linkages. Hyaluronic acid has modifiable carboxyl, hydroxyl, and amino groups to develop hyaluronic acid-based formulations [1–4]. As a vital constituent of the extracellular matrix, hyaluronic acid's structure, besides its biological features, mediates its engagement in cellular signaling and wound healing [5]. Furthermore, it is vital in cell motility and differentiation processes [6].

Hydrogels are 3-dimensional structures composed of covalently or physically linked polymeric structures, which are usually capable of holding enormous quantities of water in their network [7]. Hydrogels typically have excellent biocompatibility and can load hydrophilic substances due to their significant water content, ranging from 70–99%, thus mimicking body tissues [8]. Stimuli-responsive hydrogels are constructed to react with chemical and physical stimuli, including pH, temperature, ionic strength, light exposure, and glucose level [9, 10]. Owing to their unique characteristics, these hydrogels have been widely investigated for medical applications [11–14]. Polymers employed in the manufacture of stimulus-sensitive hydrogel systems have different interacting functional groups located throughout the primary polymeric structure; these functional groups, as well as other environmental factors, can detect the polymer configuration by affecting both polymer interactions with the solvent and interpolymeric interactions; when a polymer is in contact with a favorable solvent, interactions between segments of the polymer predominate, resulting in relaxed polymer chains. Meanwhile, the hydrogel will aggregate when it encounters a poor solvent because the chain mobility is constrained due to increased polymer-polymer interaction. Alterations in the solution's conditions, such as acidity, temperature, and ion concentration, as well as in the electrical current and ultrasonic velocity, among other external stimuli, can alter the solvent-polymer interaction [15].

### 1.1. Main Challenges Associated with Hydrogel Use in Biomedical Fields

Hydrogel delivery of hydrophobic ingredients is challenging because of its variability, inadequate solubility, poor biological stability, and difficulty controlling the release rate. However, by improving solubility and strength, it is possible to optimize the hydrogel's ability to release incorporated active ingredients in a controlled way [16, 17].

Scaling up hydrogel production for commercialization is a primary challenge in transforming hydrogel use in biomedical fields from laboratory experiments to the market [18, 19]. Scaling up hydrogel production presents a significant challenge due to the fact that hydrogel is typically made from natural polysaccharide polymers, such as hyaluronic acid. These materials are naturally driven, which makes batch-to-batch variability the primary obstacle to large-scale hydrogel production. A major obstacle in the commercialization of hydrogels is the requirement for sterilization. Traditional heat sterilization methods can lead to the destruction of hydrogel products. Alternative sterilization methods, such as gamma radiation or filtration, should be explored to overcome this challenge. In addition, hydrogel should be produced in an aseptic condition [20].

The safety of using hydrogel in biomedical applications should be carefully considered. The effects of hydrogel degradation products on normal physiological functions of body tissues remain to be studied. Hydrogel use safety might be maintained by adequately conducting the toxicological characteristics of the materials used in hydrogel manufacturing and selecting biodegradable and biocompatible materials [21].

In addition to previous limitations, the suitable mechanical property of hydrogels for human tissues is a crucial factor to consider, as this will provide an acceptable tissue reaction while avoiding injury to healthy tissues [21, 22]. The mechanical properties of hydrogels can be modulated to mimic biological tissues by controlling hydrogel crosslinking densities [23] and using metal bonds of coordination as a hydrogel crosslinker [24, 25]. Furthermore, composite hydrogels are produced by incorporating nanoparticles, which have been reported to enhance the mechanical characteristics of the hydrogel [26].

### 1.2. Injectable Hydrogels for Biomedical Applications

Injectable hydrogels used in biomedical applications are growing in popularity. The ability to be administered to the target site using an injection device is called injectable. Consequently, it is not limited to in situ gel formation but can be produced *in vivo*. After injection, injectable hydrogels must exhibit the appropriate mechanical properties [27].

Hydrogels have gained significant consideration among various matrices explored for tissue repair due to their versatility, structural resemblance to the extracellular matrix, substantial water content, biodegradability, porosity, and low cytotoxicity [28]. Over the last few years, there has been an upsurge in the investigation of injectable hydrogels in tissue regeneration because of their less invasive administration and structural flexibility [29, 30]. It is simple to perform structural modifications, modulate mechanical properties, and promote the physicochemical characteristics of hydrogels to fit different anticipated medical applications [31].

The use of hyaluronic acid hydrogel for wound healing and cancer therapy was intensively reviewed by Yang et al., indicating the significance of using injectable hyaluronic acid hydrogel in wound dressing due to its good adherence to wounds and its ability to fill wound sites [32]. The investigation of injectable gels, in general, as a localized drug delivery vehicle has attracted researchers in different disciplines [33–36], where injectable hydrogels containing chemotherapeutic drugs can be injected directly into the tumor to treat specific cancer [37–40]. Due to the injectable hydrogels' limited toxicity and the drug's localization at the treatment site, developing these pharmaceutical formulations to treat different tumors has become a research hotspot [41].

This article goes over various approaches in producing hyaluronic acid-based injectable hydrogels to aid researchers in improving their understanding of the design and development of hyaluronic acid-based injectable hydrogels for different biomedical uses.

## 2. Injectability Mechanisms

**Figure 1:**
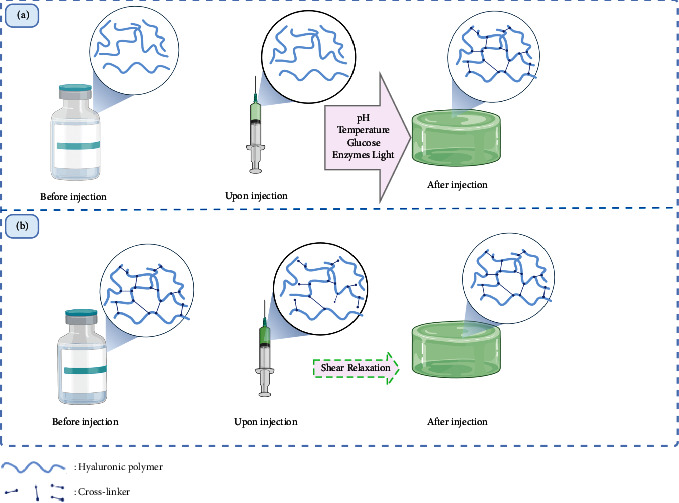
Illustration of injectability mechanisms of injectable hyaluronic acid hydrogels: (a) in situ-forming liquid and (b) shear-thinning injectable gel. This original figure was created by the authors using BioRender.com.

In terms of the injection mechanisms, the hyaluronic acid-based hydrogels can be classified into two main categories: in situ-forming injectable hydrogels and shear-thinning injectable hydrogels, as described in Figure 1.

### 2.1. Hyaluronic Acid-Based In Situ Gel-Forming Liquid

This type of hydrogel is prepared as a flowable liquid that can be converted into a hydrogel at physiological conditions triggered by various stimuli, including temperature, pH, and ionic strength, or by being subjected to other stimuli, such as light exposure [42].

#### 2.1.1. Thermoresponsive Hyaluronic Acid-Based Injectable Hydrogel

The thermoresponsive hyaluronic hydrogel was produced using a disulfide-modified poloxamer diacrylate crosslinking agent, and the in situ formed gel showed good mechanical properties with minimum swelling [43]. It has been found that hyaluronic acid modified by acetylation and glycol chitosan can interact hydrophobically to generate thermally induced gels [44]. Poly (N-isopropylacrylamide)-grafted hyaluronic acid demonstrated hydrogel formation when the heat was increased over 35°C; the formed hydrogel showed sustained release properties of riboflavin with a stable hydrogel shape after 24 hours of *in vivo* administration [45]. Another study reported the in situ formation of a hydrogel of hyaluronic poly (N-isopropylacrylamide) prepared using click chemistry [46]. Thermosensitive hyaluronic acid-based injectable hydrogels were recently reported through the conjugation of hyaluronic acid with poly (N-isopropyl acrylamide), the produced hydrogel exhibited in situ gelation at body temperature, and study results indicate good compatibility with prolonged residence at the injection site which is regarded crucial for *e* for ensuring the treatment's maximum efficacy [47].

A recorded patent claimed the formation of a thermosensitive hyaluronic acid hydrogel when combined with polypropylene and polyethylene oxide copolymers with a measured sol-to-gel transition temperature ranging from 30 to 37°C [48].

#### 2.1.2. Other Stimuli-Responsive Hyaluronic Acid-Based Injectable Hydrogel

The methacrylate-thiol Michael addition reaction resulted in light-induced gel formation of methacrylate, hyaluronic, and thiolated hyaluronic acid [49]. Figueiredo et al. used the benzoxaborin-saccharide complexation to prepare an injectable dynamic covalent hydrogel at physiological pH conditions, mainly in an epidermal application [50]. Another pH-induced hydrogel was produced using aldehyde-modified maleic sodium hyaluronate [51].

Hyaluronic acid-based injectable nanogel was prepared through salt-triggered in situ gelling; cholesteryl-conjugated hyaluronic acid was mixed with recombinant human growth hormone to obtain prolonged release properties [6].

### 2.2. Shear-Thinning Hyaluronic Acid-Based Injectable Hydrogels

Although in situ gel formation is considered a standard technique for producing injectable hydrogels, it is difficult to monitor the *in vivo* process of in situ gel formation, which limits their use in clinical applications. Shear-thinning hydrogels, which differ from in situ gelling hydrogels in that they can be created ex vivo, can change from the gel form to solution form throughout injection and reconvert directly after injection, making them appropriate for minimally invasive applications [52].

Self-healing is the process of restoring the hydrogel to its intact form. The ability of an already-formed hydrogel to flow and be injected by applying shear stress upon the use of needles and to return to the hydrogel form when relaxed after injection is referred to as shear thinning. This type of hydrogel can involve a gel formed by both chemical and physical crosslinking, and the exact mechanism of injectability differs for each hydrogel system [42, 53].

The shear-thinning hydrogel was produced using hyaluronic acid and methylcellulose to repair spinal cord damage. In addition to the shear-thinning property, the produced hydrogel indicated increased hydrogel strength with temperature increase [54].

The shear-thinning hydrogel was produced by ionic crosslinking of blended hyaluronic acid and alginate. Iron (III) metal ion coupled with ethylenediaminetetraacetic acid was evaluated as a crosslinking complex. The resultant hydrogel exhibited antimicrobial activity and could return to its gel 3D structure immediately after injection [55]. Nanofiber containing hyaluronic acid hydrogel was developed to be used as a filler for tissue regeneration applications, and the produced gel maintained the hydrogel 3D structure immediately after injection [56]. Injectable dermal fillers are one of the popular applications of this type of hydrogel. Hyaluronic dermal fillers with a soft consistency are already available on the market and are used to minimize the visible signs of facial lines [57].

## 3. Methods of Preparation

### 3.1. Crosslinking Mechanism of Hyaluronic Acid Injectable Hydrogel

Injectable hyaluronic acids are set using various physical and chemical methods to overcome the limitation of using hyaluronic acid itself [58], to prolong hydrogel residence, and to offer the hydrogel with specific mechanical, viscoelastic, solubility, degradation, and biological properties matching the clinical indications [59].

#### 3.1.1. Physical Crosslinking Mechanism of Hyaluronic Acid Injectable Hydrogel

Physical crosslinking is a simple method with low toxicity that depends on changes in different conditions, including temperature, acidity, ion type, and concentration, and it includes hydrophobic interactions, hydrogen bonding, charge interaction, and stereo complexation [60]. Few attempts to develop an injectable hydrogel based on hyaluronic acid without the addition of chemical crosslinkers or chemical modification have been reported; a physical crosslinked hyaluronic-based hydrogel usually involves blending with one or more polymers, as described in Section 3.2.

#### 3.1.2. Chemical Crosslinking Mechanism of Hyaluronic Acid Injectable Hydrogel

Most of the formulated hyaluronic-based hydrogel is crosslinked by directly including crosslinking substances or by modifying the polymeric chain to generate new functional groups that can form a hydrogel network [5]. The modifications on hyaluronic acid mainly occur at different sites: carboxylic groups, hydroxyl groups, and N-acetyl groups that can react with various functionals [61]. These modifications are performed to maintain biocompatibility and biodegradation characteristics and to form a resistant hydrogel to hyaluronidase digestion [62].

*(1) Crosslinking and Modification of the Carboxylic Acid (–COOH) Group*. The chemically crosslinked hyaluronic injectable hydrogel was formed using a chemical modification procedure for the amide linkages between the carboxyl and amino functional groups [63]. The reaction depends initially on the process of activating the carboxylic group [64], in addition to the creation of an amide or ester bond in two stages; initially, the reaction between the reactant and the carboxylic functional group of the hyaluronic acid produces a reactive moiety, the amino group of the reagent acts as the nucleophile and the nucleophile reacts with the carboxyl group and forms the ammonium hydrogel. The hydrogel prepared following these reactions was characterized by improved stability and mechanical properties [65–67].

**Figure 2:**
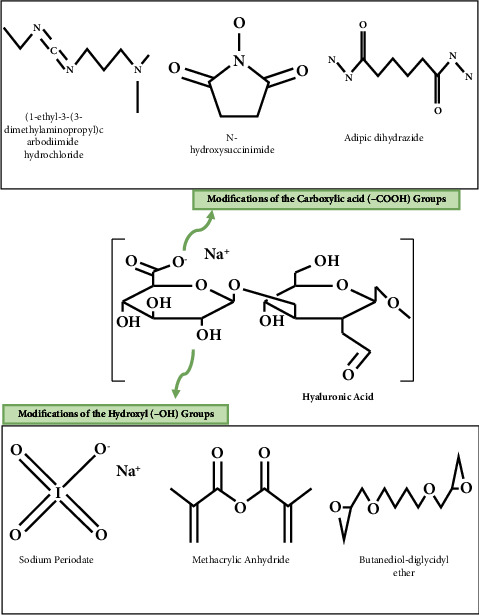
Examples of chemical modifications of the carboxylic acid (–COOH) and hydroxyl (–OH) groups of hyaluronic acid. This original figure was created by the authors.

Different chemical moieties can be used to crosslink hyaluronic acid to form in situ hydrogels, as proposed in Figure 2, which involves the use of ethyl-dimethyl-aminopropyl-carbodiimide hydrochloride and N-hydroxysuccinimide as they were reported to create ester bindings among the carboxylic and hydroxylic groups of hyaluronic acid itself or with other molecules [68, 69]. The crosslinking of hyaluronic acid with adipic dihydrazide has also been reported to sustain protein release for up to 28 days; the produced in situ-forming hydrogels were promising for bone regeneration treatments [70].

The crosslinking of hyaluronic acid was achieved using boronate esters from boronic derivatives and hyaluronic polymer binding. Crosslinking was obtained by reversible covalent bonds between hyaluronic acid, boric acid, and boronic acid derivatives [50]. This method is rapid and easy, while the hydrogel structure is more stable and flexible [71].

Hyaluronic acid modification with 3-aminophenylboronic acid has been reported for the formation of glucose-sensitive in situ-formed injectable hydrogels; this conjugation allows the transition of hyaluronic acid solution to a hydrogel that is responsive to the existence of glucose at a level that mimics the physiological concentration [72].

Another chemical modification involving the hyaluronic carboxylic group is the cycloaddition (click) Diels–Alder crosslinking, which is one of the attractive options for medical applications due to its great adaptability and good productivity [73]. Shuangli et al. produced hyaluronic acid crosslinked polyethylene glycol hydrogels by reacting with cyclooctyne-grafted hyaluronic and azide-grafted polyethylene glycol. They prepared the hydrogel injectable to be used as surgical fillers [74].

*(2) Modifications and Crosslinking of Hydroxyl Groups*. In general, modifications on hydroxyl groups include oxidation or the creation of hemiacetal, ether, or ester bonds [61].

Kenne et al. investigated butanedioldiglycidyl ether for the crosslinking of hyaluronic acid to form a hydrogel, which was performed in an alkaline pH medium to create an ether bond between the hydroxide and epoxide ring [75]. The stability of butanedioldiglycidyl ether crosslinked hyaluronic acid has been studied, indicating the formation of hydrogels with enhanced resistance toward enzymatic degradation [76]. Butanedioldiglycidyl ether crosslinked hyaluronic acid has also been investigated by Zerbinati et al. for the production of dermal fillers. The formed hydrogel was reported to have a spider-web-like structure and good elasticity [77].

Sodium periodate produces aldehydes by oxidizing hyaluronic acid hydroxyl groups, forming a linear chain by opening the sugar ring and increasing the polymeric backbone's flexibility [78]. The oxidation degree is relatively proportional to the added periodate quantity [79], and this oxidized product is considered a precursor for hydrogel fabrication using Schiff's base reactions [80–82].

The Schiff base chemical reaction occurs under physiological conditions that allow the production of in situ-forming hydrogels that are crosslinked by the reaction between amine and carbonyl groups to form a reversible imine bond; this reaction is regarded as imine formation and can be catalyzed by both acid and base catalysis [83].

This reaction produces injectable hydrogels that can be used for 3D printing with self-healing ability while maintaining its main structure [84].

These advantages of Schiff's base reactions were broadly investigated for various applications. However, the practical use of the Schiff base reaction was limited due to its pH sensitivity [32].

Ester bond formed between the anhydride of octenyl succinic anhydride and the hydroxyl of hyaluronic in alkaline conditions (pH = 9) was reported by Eenschooten et al. Hyaluronic acid degree of substitution has reached 43% in the optimal conditions [85]. Tous E. et al. investigated the formation of an ester bond between hyaluronic acid and methacrylic anhydride in alkaline media (pH: 8–10). The resulting injectable gel was reported to have good stability against enzymatic degradation; it was used in myocardial infarction cases to remodel the left ventricle and control symptomatic heart failure. This treatment reduced the myocardial wall stress from 2 to 8 weeks [86].

### 3.2. Hyaluronic Acid-Based Injectable Composite Hydrogel

Single-network hydrogels, sometimes called conventional hydrogels, have been limited when high mechanical properties are required [87]. Composites are systems of hybrid materials featuring different characteristics from the created components. The construction of a composite tries to enhance particular polymers' properties so that they are superior to those of individual polymers [88].

Hyaluronic acid-based composite hydrogels are dual network hydrogels composed of blended hyaluronic acid and other polymer/s or colloidal mixes of microparticles and nanoparticles coupled with hyaluronic acid [89].

Historically, the components of the polymeric composite have been characterized as matrices and reinforcing portions. It is crucial to consider whether the composite contains a synthetic polymer or has both a major and minor component [90]. These two phases have been given the names “matrix” continuous phase, which provides the composite material's structure and support, and “dispersed phase,” which is incorporated to enhance the matrix's properties [91]. However, it is not particularly significant for composites constructed entirely of polysaccharide polymers and may even be challenging to determine because polysaccharides exhibit comparable surface characteristics [92].

#### 3.2.1. Hybrid Hyaluronic Acid-Based Injectable Hydrogels

In general, polymeric blending is used in hydrogel formulations to enhance hydrophilicity [93, 94], promote antimicrobial activity [95], improve compatibility, and modulate mechanical properties [96–98]. The diversity of hyaluronic acid-based injectable hydrogels is being expanded by hybrid hydrogels, in which several polymers with different physicochemical properties are combined in a single unit and show significant modifications in their mechanical properties, which reflect on their release properties, drug delivery targeting, and overall strength and durability of the system [89].

The preparation of polymeric blends is considered a simple method to get the desired polymeric properties from each polymeric component [99]. Polymeric blending in the preparation of hydrogels is considered a typical method to produce different materials that exhibit many features that cannot be achieved by utilizing each polymer alone. In general, the materials used in the hydrogel preparation are principally considered by the presence of multiple functional moieties, leading to the implementation of different modifications. For instance, combining synthetic and naturally driven materials allows tuning the physicochemical properties of materials to merge the biocompatibility and safety of natural polymers with the different favorable physicochemical properties of synthetic ones [87, 100].

In most cases, blending of hyaluronic acid with other polymers for the preparation of injectable hydrogels aimed to promote mechanical properties of the hydrogel, as reported for a hyaluronic blend with polyethylene glycol [74], oxidized pectin [101], glycol chitosan [102], poly (*γ*-glutamic acid) [103, 104], and gelatin [105]. Other studies use polymeric blends with hyaluronic acid to improve the gel's stability and slow its degradation, as reported using gelatin [106] and glycol chitosan [102]. Blending with hyaluronic acid was also used to promote cell adhesion, as reported for fibrinogen with hydroxyphenyl-modified hyaluronic acid to obtain stem cell adhesion [107]. Adhesive properties were also obtained using gelatin blended with tyramine-modified hyaluronic acid [105]. Hyaluronic acid is also blended with pluronic acid and cyclodextrin polymers to modify the hydrogel's rheological characteristics [108]. Furthermore, methylcellulose was blended with hyaluronic acid to gain fast gelling properties, as the gel formed within two minutes when incubated at 37°C without the need of using a crosslinker or chemical modification [54].

Two primary methods for polymer blending are solution blending [109] and fusion blending [110]. Solution blending is the most applicable method, which is simple and suitable for preparing several applicable forms [111]. Solution blending can be done by dissolving each polymer in a suitable solvent and mixing the two polymeric solutions; in addition, the crosslinker can be added to improve the mechanical strength [112, 113]. The properties of polymeric blends are determined by potential interactions between two polymers [114].

*(1) Chemically Crosslinked Hyaluronic Acid-Based Hybrid Injectable Hydrogel*. Preparing a hybrid in situ-forming hyaluronic acid injectable hydrogels usually requires chemical modification of hyaluronic acid, as represented in Table 1, to allow chemical crosslinking between two polymers. The hyaluronic acid can be conjugated with thiol and hydrazide to permit double crosslinking with oxidized sodium alginate through the formation of hydrazone bonds and disulfide bonds [115]. Hydrazone bond-based crosslinking was also reported for thiolate-modified hyaluronic acid with acidic type I collagen [117]. Hyaluronic acid can also be oxidized to form an aldehyde-modified hyaluronic acid upon cleavage; this aldehyde group can react with amine-containing proteins and polymers, which can allow the chemical crosslinking through the formation of imine bonding; this reaction called Schiff's base reaction and used to covalently combine hyaluronic acid with different derivatives of chitosan polymer including N-succinyl-chitosan [119], N, O-carboxymethyl chitosan [118], carboxymethyl chitosan [116], and gycol chitosan [102]; Schiff's base reaction also reported for oxidized hyaluronic with carbohydrazide groups of the carbohydrazide-modified gelatin [106]. A chemical crosslinking agent such as genipin can prepare hyaluronic combined with collagen and chitosan polymers [120].

Photopolymerization of methacrylate hyaluronic acid when combined with polyglutamic acid [103] and heparin [122], self-healing maleic hyaluronic acid-based-hydrogel is produced through acyl hydrazone bonding combined with photocrosslinking due to the aldehyde modification of the maleic hyaluronic salt, the produced hydrogel was with good cytocompatibility, mechanical strength, and pH responsiveness [127].

Enzymatical crosslinking can also obtain injectable hyaluronic-based hydrogels by forming hydroxyphenyl-modified hyaluronic acid's enzymatically induced soft hydrogel when combined with fibrinogen [107]. Another study reported the enzymatic crosslinking of tyramine-modified hyaluronic acid and gelatin as the resulting phenolic group reacted with the peroxidase enzyme to form the hydrogel [105].

*(2) Physically Crosslinked Hyaluronic Acid-Based Hybrid Injectable Hydrogel*. Several attempts have been made to obtain physically crosslinked hyaluronic acid-based injectable hydrogels without chemical agents; hyaluronic acid combined with poloxamer and k-carrageenan was reported to form a physical hydrogel [123, 124]. However, in both studies, poloxamer was the foremost hydrogel component, and hyaluronic acid was added in minor concentrations.

Blending two anionic polymers will affect the swelling ability of the prepared gel by varying the exterior pH conditions. In the presence of the anionic, carboxylic, or sulfate moieties, increasing pH is expected to increase the polymeric chain charge, which then leads to an increase in repulsive forces along the polymer chains, which then results in the formation of an expanded network; this extreme pH-dependent behavior explains the potential of using this type of polymeric combination for the construction of pH-responsive hydrogels [51]. This explains the promoted swelling and drug release of hybrid hyaluronic carrageenan and poloxamer hydrogels [123]. Physical crosslinking was also reported with fish gelatin [128] and gellan gum [129].

Hydrogels can be produced when two polyelectrolytes with opposing charges are mixed; the electrostatic interaction of two opposed charges is primarily responsible for the production of polyelectrolyte complexes, causing interpolymeric ion condensation and the release of counterions as a result [130–132]. H-bonding, ionic, or hydrophobic forces maintain this hydrogel structure [133]. Since all of these interactions are reversible, they might be destroyed by altering the surrounding conditions [134]. The formation of a hydrogel of hyaluronic acid with chitosan and chitosan derivatives has been studied [44, 126]; Vignesh et al. reported the development of the coacervation complex by nonspecific electrostatic binding with the chitosan polymer [125]. Thermally induced gelling through the formation of hydrophobic interactions between N-hexanoylation of glycol, chitosan, and hyaluronic acid modified by acetylation followed by additional physical crosslinking has been reported [44].

#### 3.2.2. Hyaluronic Acid-Based Injectable Hydrogels Incorporating Nanoparticles

Significant efforts have been carried out in recent years to create innovative drug delivery technologies that incorporate drug-loaded nanoparticles into injectable hydrogels, as this allows site-specific delivery of drugs targeting tumor cells [135–137]. Hydrogel nanoparticles are one of the attractive medication delivery approaches as these systems merge the functionalities of hydrogel and nanoparticles; they allow for localized and targeted delivery of the drug combined with properties of small particles and controlled release properties [138, 139].

**Figure 3:**
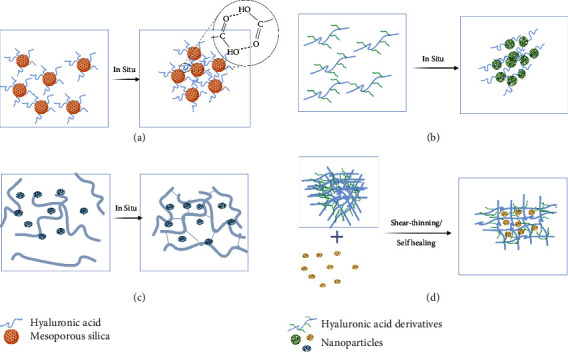
Simple illustration of various design approaches of hyaluronic acid injectable nanocomposite hydrogel: (a) nanoparticles containing hyaluronic acid in the surface shell that aggregated in situ to produce hydrogels, (b) hyaluronic derivative-based hydrogels designed with the capability of in situ-forming nanoparticles, (c) nanoparticles mixed with chemically modified hyaluronic acid that enable to in situ form hydrogel, and (d) nanoparticles act as a node to crosslink the hydrogel triggering the formation of shear-thinning composite hydrogel. This original figure was created by the authors using BioRender.com.

As illustrated in Figure 3, hyaluronic acid injectable hydrogels incorporating nanoparticle technology can be designed using different strategies. First, nanoparticles containing hyaluronic acid in the surface shell that aggregated in situ to produce hydrogels as constructed by Chen et al. in their study on the preparation of a tumor-targeting nanocapsule formulation composed of a hyaluronic acid shell and mesoporous silica-based core, besides targeting the CDD4 receptor on the tumor cells surface, showed a pH sensitivity as the low pH of the tumor environment triggers the in situ gelation of hyaluronic acid [140].

More recently, hyaluronic derivative-based hydrogels were designed with the capability of in situ-forming nanoparticles; the transformation to nanoparticles was achieved at a temperature above 30 C; the production involved the use of poly-N-isopropyl acrylamide and sulfo-dibenzocyclooctyne-PEG4-amine; the produced hydrogel system has a prolonged residence time and superior stability toward both enzymatic and oxidative degradation [141].

Nanoparticles mixed with chemically modified hyaluronic acid can form hydrogels in situ. Hu et al. demonstrated the production of a composite hydrogel composed of chemically crosslinked hyaluronic acid through in situ polymerization, and this hydrogel is embedded with a pH-sensitive nanoparticle for cell therapy applications. The composite hydrogel showed a pH sensitivity with good mechanical characteristics and was superior to the corresponding noncomposite hydrogel [142]. Hyaluronic acid hydrogel incorporating drug nanocrystals provides a potential intraarticular treatment approach for inflammatory arthritis, taking advantage of the depot formation of the hydrogel combined with the release, solubilization, and stabilization advantages of drug encapsulation as a nanocrystal [143].

Besides the typical advantages of encapsulating drugs in nanoparticles, such as improving drug solubility, stability, and release characteristics, nanoparticles can also act as a node to crosslink hydrogels, presenting an innovative approach in developing nanocomposite hydrogels. Hybrid thiol-modified hyaluronic hydrogel incorporating silver-lignin nanoparticles, where the nanoparticles act as a node to crosslink the hydrogel, resulted in a composite hydrogel that has a shear-thinning ability and was found to be promising for chronic wound healing treatments [144].

While preclinical investigations demonstrate the advantages of incorporating nanoparticles into hydrogel products, the clinical translation of nanomedicines has proved complicated [145].

## 4. Biomedical Applications of Injectable Hyaluronic Acid-Based Hydrogel

### 4.1. Injectable Hyaluronic Acid-Based Hydrogel for Tissue Engineering Applications

Tissue engineering principally involves the use of synthetic as well as natural tissue analogs to restore or replace destroyed or deteriorating tissues. Generating innovative tissue engineering scaffold materials with superior characteristics is especially important.

Injectable hydrogels are among the most intriguing biomedical products that can be employed in tissue engineering applications, and their use is advantageous as they can be injected directly into the area of the injury without the need for surgery. Besides, hydrogels can be easily loaded with cells and active molecules and might control their delivery in vivo. Furthermore, the injectable hydrogel can be shaped within the body to attain the shape of the injection area. It can supply the cells with a proper three-dimensional surrounding environment analogous to their naturally situated within the tissues' extracellular matrices [42, 146]. Injectable hydrogels have been utilized to regenerate cartilage [147], bones [147, 148], skin [149], heart tissues [150], and nervous system tissues [151]. Reported injectable hydrogel systems based on hyaluronic acid for tissue regeneration are summarized in Table 2.

Hyaluronic acid-based hydrogels are attractive for tissue engineering applications as they are nonimmunogenic, biocompatible, and biodegradable and are a primary extracellular matrix element of connective tissues [153, 154].

Injectable hydrogels consisting of oxidized hyaluronic acid covalently linked with carboxylated chitosan was investigated as a vitreous replacement by Wang et al. *In vivo* experiment results indicated the ability of the produced vitreous substitute to retain the retina at its position and keep the ocular pressure without any considerable side effects [116].

The hybrid injectable hydrogels of aldehyde hyaluronic acid and carboxymethyl chitosan with good biocompatibility and cytocompatibility, exhibiting anti-inflammatory and tissue regeneration capabilities when employed for abdominal cavity defects, the addition of chitosan derivatives and the formed imide linkage strengthen and slow down hyaluronic acid degradation to mimic the rate of abdominal tissue regeneration [118].

#### 4.1.1. Injectable Hyaluronic Acid-Based Hydrogel for Articular Cartilage Repair

Phan et al. developed a porous injectable hydrogel for articular cartilage repair; the constructed hydrogel was composed of silk fibrin combined with hyaluronic acid crosslinked by Schiff's base reaction. Furthermore, glutaraldehyde and ultrasonication were used to trigger in situ gel formation, and methylprednisolone was loaded in the produced gel as an active ingredient to lower inflammatory response. The developed hydrogels were found to be able to control methylprednisolone release as a result of their slow biodegradation characteristics. Besides, *in vivo* experimental evaluation indicates the potential of using the constructed hydrogel for cartilage regeneration [152]. Another study reported the investigation of injectable hydrogels for articular cartilage tissue repair; the developed hydrogel consisted of thiolated hyaluronic acid and collagen, and *in vivo* evaluation indicated an enhancement in cartilage formation when using this hydrogel system [117]. A hybrid hydrogel system of hyaluronic acid, chitosan, and methylcellulose derivatives was also evaluated for cartilage tissue repair and found to increase the rate of tissue repair and enhance the matrix deposition and proliferation; the developed hydrogel can be easily produced by physical blending with no chemical crosslinker addition [126].

#### 4.1.2. Injectable Hyaluronic Acid-Based Hydrogel for Neural Tissue Engineering Applications

Hyaluronic acid-based injectable hydrogel systems were investigated for neural tissue engineering applications. For instance, hydroxyphenyl-modified hyaluronic acid has been used for the construction of injectable hydrogels for the regeneration of spinal cord defects; the chemically modified hyaluronic acid was blended with fibrinogen and embedded with mesenchymal stem cells, and fibrinogen protein was added to improve cell migration and adhesion, and proliferation. *In vivo* evaluation of a partial defect in the spinal cord indicated the prospect of using this hydrogel system for tissue regeneration. Besides the capability to encapsulate stem cells, the hydrogel covers the injured cavity and promotes axonal sprouting and vascularization [107]. The self-healing hydrogel of hyaluronic acid and methylcellulose polymers was developed and evaluated for spinal cord tissue repair; the produced hydrogel exhibited thixotropic rheological behavior, explaining its good injectability combined with the thermal gelation effect of methylcellulose that suggested the potential of its investigation for sustained delivery applications, besides that, the produced hydrogel system exhibited no cell adhesion, biodegradability, and biocompatibility inside the intrathecal space [54].

#### 4.1.3. Injectable Hyaluronic Acid-Based Hydrogel for Bone Tissue Regeneration Applications

For osteoarthritis defect repair, an injectable hyaluronic and heparin hydrogel was assessed for the local delivery of two types of transforming and platelet-derived growth factors; a microfluidic technique with the assistance of photopolymerization was used to formulate the microgel, compared to the conventional treatments, the developed injectable microgel suggested to be promising for osteoarthritis restoring as the study indicated an improvement in cell adhesion, migration, and differentiation [122].

Injectable hydrogel loaded with osteogenic compound graphene oxide was reported to be promising for bone tissue regeneration applications; the produced hydrogel was able to encapsulate and deliver the active ingredient directly to the defect site in a controlled dose [102].

A thermoresponsive and ultrasound-sensitive injectable hydrogel, composed of hyaluronic acid and a pluronic and gelatin composite system, has been recently developed. Both in vivo and in vitro examinations of the hydrogel have shown its potential for delivering hydrocortisone to treat osteoarthritis. Nevertheless, hydrocortisone release is triggered by ultrasound and also has pH-dependent release [155].

### 4.2. Wound Healing Applications

Skin wound repair and healing is a complex process containing a combination of several cellular and matrix elements at multiple stages [156]. During the initial phase, inflammatory cells such as macrophages gathered and released high amounts of chemokines, attracting functioning fibroblasts and repairing the damaged area by depositing collagen fibers [157]. Late in the process, the fibroblast-driven matrix promotes vasculature formation in a three-dimensional environment and nourishes the buildup of granulation tissues [158]. Since fibroblasts perform an essential role, the speed at which they migrate to the injury site significantly affects how quickly a wound heals. In addition, growing evidence suggests that the mechanical properties of scaffolding materials may influence cell proliferation and migration since it may alter the mechanical transduction in the interfaces [104, 159].

The hydrogel-based wound dressing is gaining popularity due to its benefits in establishing a three-dimensional environment for cell attachment, immigration, and proliferation [160]. In particular, the injectable hydrogel used in wound healing is an area of interest because it can adhere to a lesion's erratic shape while providing a vehicle for drug and cell delivery [36, 161, 162].

Several scientists have constructed various hyaluronic acid-based hydrogels and investigated the capabilities of the developed hydrogel for wound healing. The previous article reviews the reported use of hyaluronic hydrogels as a wound dressing [32].  Table 3 summarizes the recent publications on injectable hyaluronic hydrogels for the healing and repairing of acute, diabetic, and chronic wounds.

A more recent study reported the investigation of thiol and catechol-modified hyaluronic polymer for diabetic wound healing applications. Conjugated hyaluronic acid polymer is combined with polyhexamethylene guanidine and black phosphorus, and the resulting hydrogel of this combinatiob has good self-healing properties due to the dynamic nature of chemical coupling between the polymeric structures. When compared to commercially available wound dressings, in vivo experiments revealed that the developed hydrogel exhibited improved and accelerated healing properties. These properties were explained by the hydrogel's antibacterial, antioxidant, and anti-inflammatory properties as well as by the observed increase in granulation tissues and collagen deposition [176]. Han et al. recently reported the production of a physically crosslinked hydrogel composed of oxidized hyaluronic acid combined with tannic acid and quaternary chitosan; the produced hydrogel displayed good healing properties as indicated by *in vivo* experiments, and this effect was attributed to the intrinsic antioxidant and antibacterial characteristics of the forming polymers [177].

Bioadhesive hydrogel having inherent antimicrobial activity was produced by combining *ε*-polylysine functionalized hyaluronic acid that double crosslinked via Schiff's base reaction and by peroxidase enzymatic crosslinking, the developed hydrogel displayed antimicrobial action against different bacterial species; the antibacterial action mainly related to the amine group of *ε*-polylysine. *In vivo* evaluation of the treatment of wounds on rats displayed accelerated wound healing, and the study indicated two-fold enhancement in the growth of new skin, micro vascularization, collagen, and tissue granulation compared to the commercially available fibrin sealant [163]. Another study reported the construction of hyaluronic acid-based injectable hydrogels with antibacterial and wound-repairing capabilities created using aldehyde hyaluronic acid covalently bound to hyaluronic acid grafted with adipic acid and combined with sisomicin sulfate. The produced in situ formed hydrogels displaying pH and enzymatic sensitivity; this study suggested the significance of using the studied hydrogels for their wound healing and antibacterial activities as the release of sisomicin sulfate from the hydrogel is expected to increase at acidic pH due to the indicated higher degradation rates in acidic media which allows the on-demand release in wound site with bacterial infection. Besides that, the study results indicated that the increased sisomicin sulfate level in the hydrogel reduces the inflammatory response in the wound process [164].

### 4.3. Drug and Cell Delivery Applications

The typical systemic administration of active ingredients is accompanied by undesirable adverse effects [178–181]. Therefore, an alternative to the systemic parenteral route of administration is local site-specific delivery systems, as they enable the medication to be localized at the site of action with a lower dose. Therefore, these delivery systems are expected to exhibit minimal systemic absorption and limited undesired side effects and drug-drug interactions [182].

#### 4.3.1. Injectable Hyaluronic Acid-Based Hydrogel for Drug Delivery Applications

Drug-loaded injectable hydrogels can effectively minimize systemic side effects by releasing drugs locally at the site of the tumor. Due to the injectable hydrogels' limiting toxicity and the drug's localization at the action site, developing these pharmaceutical formulations to treat different malignancies has become a research hotspot [37, 41]. Besides that, drug release from a hydrogel matrix could be controlled by a variety of mechanisms [183, 184].

The rate at which active ingredients are released from the hydrogel may be changed advantageously in response to external factors, if the crosslinking process is reversible, or if the hydrogels incorporate stimuli-responsive moieties in their structural components [27].

Zhang et al.'s study stated the in situ double crosslinking to produce an injectable hydrogel, which was constructed using hyaluronic acid featured by thiol and hydrazide moieties added to oxidized alginate. This hydrogel displayed a considerable ability to be used as a drug carrier, as the release studies indicated extended-release characteristics of the model drug—bovine serum albumin—from the developed hydrogel system [115].

Self-healing hyaluronic acid combined with carboxymethyl chitosan hydrogel has been recently reported to sustain the release rate of acetylsalicylic acid as a model drug. The injectable hydrogel was produced with proper mechanical strength through Schiff's base reaction, and study results indicated the potential of using this hydrogel for drug delivery applications as it can encapsulate high amounts of active ingredients and prolong drug release with pH-dependent properties combined with inherent hydrogel antibacterial properties [185].

Sustained release properties of 5-fluorouracil were obtained for the loaded hydrogels composed of hyaluronic acid, k-carrageenan, and poloxamer polymers; study results indicated that the drug release was determined by diffusion, and accumulative drug amounts of 60% were released in the first four hours meanwhile another 20% of the drug took three days to be released. In addition, the optimized hydrogel was highly elastic and thermosensitive with antiadhesive properties [124].

Berberine-loaded supramolecular bioadhesive injectable hydrogels were constructed using a polymeric mixture of hyaluronic acid and carboxymethyl-hexanoyl chitosan, then effectively encapsulated with berberine, the study indicates prolonged and pH-responsive drug release from the developed hydrogels, suggesting the potential of using these injectable hydrogel systems for biomedical applications [186].

Vignesh et al. reported the production of hyaluronic hydrogels formed through nonspecific electrostatic binding with cationic chitosan polymer reported with the ability to encapsulate deferoxamine-containing PLGA nanoparticles for therapeutic angiogenesis applications, drug-loaded nanoparticles addition did not disturb the smooth injectability of constructed hydrogels, besides that, the dissolution studies indicated a controlled release property of deferoxamine from this delivery system [125].

#### 4.3.2. Injectable Hyaluronic Acid-Based Hydrogel for Cell Delivery Applications

Injectable hydrogel prepared by photocrosslinking of copolymeric hyaluronic acid with poloxamer was reported for the delivery of bovine chondrocytes; the produced in situ-forming hydrogel exhibiting slow release rate of the loaded cells suggested as a promising candidate for cell delivery applications [121]. Another study reported the effectual cell delivery of hyaluronic acid-based injectable hydrogels produced from chemically crosslinked hyaluronic acid through hydrazone crosslinking blended with oxidized pectin. The developed hydrogel is suggested to be gradually destroyed by glutathione formed by the cells, allowing sufficient space for cell proliferation [101, 187].

Mesenchymal stem cells embedded in hyaluronic acid, chitosan, and glycerophosphate systems for treating myocardial infarction. *In vivo* experiments indicated the improved therapeutic effectiveness of mesenchymal stem cells when loaded in the developed hydrogel, as the study displayed enhancement in cardiac function and vascularization associated with lowered cell apoptosis [188].

## 5. Conclusions and Outlooks

This article provides a summary of injectable hydrogels prepared using hyaluronic acid. When used as a principal constituent of injectable hydrogels, the distinctive properties of hyaluronic acid attracted researchers from different disciplines to fabricate and evaluate hyaluronic-based injectable hydrogels using different strategies. It can be noted that researchers primarily intended to increase the mechanical strength of hyaluronic acid to be more suitable for medical use. Most of the discussed hydrogels exhibit in situ gel formation under physiological conditions, and there have been several attempts to produce hydrogels with shear-thinning ability. More studies are required to understand the techniques used to produce this class of hyaluronic hydrogels.

Most of the produced hydrogels involve chemical modifications to the hyaluronic acid polymer. Limited attempts were reported to produce the physically crosslinked hyaluronic acid injectable hydrogel. More focus has recently been noted on the production of composite hydrogels, either by combining hyaluronic acid and other materials to modulate the hydrogel's mechanical characteristics or by including nanoparticles to improve both gel strength and encapsulated active molecules release and targeting properties.

Incorporating nanoparticles in hydrogels will lead to the design and manufacturing of superior products with active component stabilization, solubilization, and optimization of release properties previously unattainable by intact hydrogels; this introduces various issues for researchers interested in the investigation of hydrogel biomedical applications.

Hyaluronic injectable hydrogels were widely investigated and found to be promising in different biomedical fields, including tissue regeneration, drug and cell delivery, and wound healing. Despite numerous preclinical studies on injectable hydrogels in biomedical applications, further research is needed to determine how the hydrogel's breakdown products affect the regular physiological activities of body tissues. Biomedical research primarily focuses on clinical translation, and scaling up hydrogel production is required to remove barriers hindering their real-world application in various medical fields.

Overall, it is worthwhile investing more effort in the design strategies for manufacturing hyaluronic acid-based injectable hydrogels to enable rapid translation into clinical use, especially given the significant potential of this hydrogel. Furthermore, collaborative research bringing together specialists from different fields will be crucial for more effective hydrogel implementation in biomedical fields.

## Figures and Tables

**Table 1:** The polymeric composition, type of chemical modification or crosslinking method, and gelation time of an injectable hybrid hydrogel-containing hyaluronic acid.

Blended polymer	Modification of hyaluronic acid	Crosslinking mechanism	Gelling time in physiological conditions at 37 C	Ref
Oxidized sodium alginate	Thiol and hydrazide	Hydrazone bonds and disulfide bonds	157–955 seconds	[115]
Polyethylene glycol	Cyclooctyne	Cycloaddition click reaction	5–50 minutes	[74]
Carbohydrazide-modified gelatin	Oxidized	Schiff base reaction	1–6 seconds	[106]
Oxidized pectin	Adipic dihydrazide	Hydrazone	112–399 seconds	[101]
Glycol chitosan	Oxidized	Schiff base reaction	18–47 seconds	[102]
Carboxymethyl chitosan	Oxidized	Schiff base reaction	60–360 seconds	[116]
Acidic type I collagen	Thiol	Disulfide bond	—	[117]
N, O-carboxymethyl chitosan	Oxidized	Schiff base reaction	70 s–2400 seconds	[118]
N-succinyl-chitosan	Oxidized	Schiff base reaction	—	[119]
Collagen and chitosan	—	Genipin crosslinking agent	—	[120]
Pluronic F-127	—	Photo crosslinking	—	[121]
Heparin	Methacrylate	Photopolymerization processes	—	[122]
Polyglutamic acid	Methacrylate	Photopolymerization	—	[103]
Polyglutamic acid	Glycidyl methacrylate-conjugated oxidized hyaluronic acid	Labile hemithioacetal bonds + photocrosslinking	17–210 seconds	[104]
Fibrinogen	Hydroxyphenyl	Enzymatical crosslinking	61 ± 4 seconds	[107]
Gelatin	Tyramine	Enzymatical crosslinking	4–9 minutes	[105]
*κ*-carrageenan and pluronic F-127	—	Physical crosslinking	6–15 seconds	[123]
Poloxamer, *κ*-carrageenan	—	Physical crosslinking	51–72 seconds	[124]
Chitosan	—	Physical crosslinking/complex coacervation	1-2 h	[125]
Chitosan		Physical crosslinking	3 h	[126]
N-hexanoylation of glycol chitosan	Acetylation	—	—	[44]
Poloxamer and cyclodextrin	—	Physical crosslinking	—	[108]

**Table 2:** Injectable hydrogel systems based on hyaluronic acid: polymeric composition, encapsulated ingredients, crosslinking method, defected tissue type, and injectability mechanism.

Blended Polymer/s	Active ingredient/s	Crosslinking type	Tissue type	Injectability	Ref
Hyaluronic acid **s**ilk fibroin	Methylprednisolone	Chemical	Cartilage	In situ	[152]
Hyaluronic acid acidic type I collagen		Chemical	Cartilage	In situ	[117]
Hyaluronic acid chitosan and silanized-hydroxypropyl methylcellulose	Chondrocytes	Physical	Cartilage	In situ	[126]
Hyaluronic acid-adipic dihydrazide oxidized pectin		Chemical	Cartilage	In situ	[101]
Acetyle hyaluronic acid and glycol chitosan	Chondrocytes	Physical	Cartilage	In situ	[44]
Hyaluronic acid and methylcellulose	—	Physical	Spinal cord	Self-healing	[54]
Hydroxyphenyl-modified hyaluronic acid and fibrinogen	Mesenchymal stem cells	Enzymatic	Spinal cord	In situ	[107]
Oxidized HA carboxymethyl chitosan	—	Chemical	Vitreous	In situ	[116]
Methacrylate hyaluronic acid and heparin	Growth factors	Photopolymerization	Osteoarthritis	—	[122]
Oxidized hyaluronic and glycol-modified chitosan	Graphene oxide	Chemical	Bone	In situ	[102]
Aldehyde-modified hyaluronic and carboxymethyl chitosan	—	Chemical	Abdominal wall	In situ	[118]

**Table 3:** Injectable hydrogel systems based on hyaluronic acid for wound healing applications: polymeric composition, encapsulated ingredients, crosslinking method, investigated wound classification, injectability mechanism, and indicated functional characteristics explained the potential of the constructed hydrogels for wound healing applications.

Components	Active ingredients	Crosslinking type	Application	Injectability	Function	Ref
Oxidized hyaluronic acid and *ε*-polylysine		Chemical and enzymatic	Acute	Self-healing	Antibacterial and accelerated wound healing	[163]
Aldehyde-modified hyaluronic acid and adipic acid dihydrazide-modified hyaluronic acid	Sisomicin sulfate	Chemical	Acute	In situ	Antibacterial, antioxidant, and hemostatic effect	[164]
Poly-*γ*-glutamic acid and oxidized hyaluronic acid		Chemical and photocrosslinking	Acute	In situ	Promote collagen deposition and increase vascularization	[104]
Hyaluronic acid blended with *κ*-carrageenan and pluronic F-127	Meropenem	Physical	Chronic	In situ	Antibacterial drug release, migration, adhesion, and cell proliferation	[123]
Furfurylamine-modified hyaluronic acid blended with maleimide polyethylene glycol		Physical	Acute	Self-healing	Antibacterial anti-inflammatory	[165]
Dopamine-modified hyaluronic acid		Chemical and physical	Acute	In situ	Antibacterial Good hemostasis and absorb tissue exudates	[166]
Dopamine-modified hyaluronic acid combined with reduced graphene oxide		Chemical	Acute	Self-healing	Antioxidant antibacterial	[167]
Methacrylate hyaluronic acid	Hydrogen sulfid**e**	Chemical	Acute	In situ	Increase re-epithelialization, cell proliferation, collagen deposition, and angiogenesis	[168]
Tyramine-modified hyaluronic acid and hydroxybenzoic-modified collagen		Chemical	Acute	In situ	Increase re-epithelialization, vasculature, and collagen deposition	[169]
Thiolate-modified hyaluronic acid and acrylate-modified polyethylene glycol		Chemical	Diabetic	In situ	Anti-inflammatory increase re-epithelialization and vasculature	[170]
Oxidized hyaluronic acid, quaternary ammonium-modified chitosan, and kalium *γ*-cyclodextrin	*α*-lipoic acid	Chemical	Chronic	Self-healing	Antibacterial and antioxidant-enhanced tissue granulation and collagen deposition	[171]
Aldehyde-modified hyaluronic acid and carboxyethyl chitosan		Chemical	Diabetic	Self-healing	Anti-inflammatory increase re-epithelialization, vasculature, collagen deposition, and cell proliferation	[172]
Oxidized hyaluronic acid and antimicrobial peptide		Chemical	Chronic	Self-healing	Antibacterial	[173]
Diphenylalanine-modified hyaluronic acid and curcumin		Chemical	Diabetic/chronic	Self-healing	Prolonged release of curcumin	[174]
Phenylboronate conjugated hyaluronic acid and quaternary modified chitosan	Magnesium	Physical	Diabetic/chronic	Self-healing	Anti-inflammatory increase vasculature, collagen deposition, and granulation tissue	[175]
Thiolate-modified hyaluronic loaded	Silver-lignin nanoparticles	Physical	Diabetic/chronic	In situ	Antioxidant, antibacterial, and anti-inflammatory	[144]
Thiol- and catechol-modified hyaluronic acid polyhexamethylene guanidine and black phosphorus nanosheets		Chemical	Diabetic/chronic	Self-healing	Antibacterial and anti-inflammatory increase collagen deposition and granulation tissues	[176]
Oxidized hyaluronic acid, tannic acid, and quaternary chitosan		Physical	Chronic	Self-healing	Antioxidant and antibacterial	[177]

## Data Availability

The data supporting this review are from previously reported studies and datasets, which have been cited. The processed data are available from the corresponding author upon request.
